# Effect of dietary oridonin supplementation on growth performance, gut health, and immune response of broilers infected with *Salmonella pullorum*

**DOI:** 10.1186/s13620-018-0128-y

**Published:** 2018-07-31

**Authors:** Qiu Jue Wu, Xiao Chuan Zheng, Tian Wang, Tie Ying Zhang

**Affiliations:** 1 State Key Laboratory of Animal Nutrition, Institute of Animal Sciences of Chinese Academy of Agricultural Sciences, NO.2, Yuan Ming Yuan West Road, HaiDian District, Beijing, 100193 People’s Republic of China; 2 College of Animal Science and Technology, Henan University of Science and Technology, No. 263, Kaiyuan Road, Luoyang, 470003 Henan People’s Republic of China; 3 College of Animal Science and Technology, Nanjing Agricultural University, No. 6, Tongwei Road, Xuanwu District, Nanjing, 210095 Jiangsu People’s Republic of China

**Keywords:** Broilers, Gut morphology, Immune, Intestinal microbiota, Oridonin

## Abstract

**Background:**

The effects of dietary supplementation of oridonin (ORI) on growth performance, cecal microbiota, epithelium development and antioxidant and immune parameters of broilers infected with *S. pullorum* were studied. A total of 300 1-d-old male chicks were selected and divided into 5 trial groups (6 replicates of 10 chickens): 1) nonchallenge control chicks (**CON**), 2) chicks treated with Salmonella Challenged Control (**SCC**), 3) chicks treated with *S. pullorum* challenge and 50 mg/kg ORI (O**1**), 4) chicks treated with *S. pullorum* challenge and 80 mg/kg ORI (O**2**), and 5) chicks treated with *S. pullorum* challenge and 100 mg/kg ORI (O**3**).

**Results:**

The results showed that *S. pullorum* had no effect on the feed intake (FI), body weight gain (BWG) or feed conversion ratio (FCR) of broilers compared with the values measured for the CON group (*P* > 0.05). However, compared with the characteristics of CON, *S. pullorum* showed effects on the counts of *Salmonella* and *Lactobacillus* at 7 d and at 14 d (*P* < 0.05), on jejunal development at 7 d (*P* < 0.05), and on jejunal immunoglobulin A (IgA) concentration at 7 d (*P* < 0.05). The addition of 100 mg/kg ORI had the greatest effect on the counts of *Lactobacillus* and *Salmonella* in cecal content (*P* < 0.05), malonaldehyde (MDA) content in the jejunum (*P* < 0.05), villi height of the small intestine, and IgA concentrations in the jejunum (*P* < 0.05).

**Conclusions:**

The results suggest that ORI can improve *Salmonella*-induced immune responses and protect intestinal health, not only through its immune inhibitory properties but also through its multi-protective effects on gut health.

## Background

*S. pullorum* is a capnophilic gram-negative rod-shaped bacterium that can cause pullorum disease (PD). Pullorum disease was once enzootic in many areas of the world [1]. Broiler chickens are known to be extremely sensitive to *S. pullorum* infections in the first 7 d of their life because of delayed development and establishment of their normal intestinal flora. *S. pullorum* infection can cause nutritional (protein, amino acid, and vitamin) deficiency, intestinal flora disorder, reduction in production performance, and invasion of internal organs in young birds, resulting in significant economic losses to the poultry industry [2]. Therefore, several synthetic chemicals (such as zinc-bearing clinoptilolite) have been used to control or limit the intestinal colonization and invasion of *S. pullorum* in poultry production [3].

*Isodon rubescens* (Donglingcao in Chinese) is widely used in traditional Chinese medicine and has long served as a popular medicine for respiratory and gastrointestinal bacterial infections, inflammation, and cancer [4]. Oridonin (ORI) is claimed to be a valid natural compound and one of the richest ent-kaurane diterpenoids of *Isodon rubescens*. ORI has generated much interest because of its notable pharmacological and biological activities, such as its anti-bacterial and anti-inflammatory properties and its ability to control viral replication, eliminate active oxygen radicals, and protect other internal organs, among other abilities [5]. Substantial research efforts have shown that isolated oridonin is used alone or in combination with other drugs to prevent and cure bacterial infection in vivo and in vitro, and since 1976, oridonin has been known to exhibit antimicrobial activity against gram-positive bacteria [6]. However, to the best of our knowledge, few studies have focused on the effects of dietary supplementation of oridonin or derivatives on antimicrobial activity against *S. pullorum* in animals, with no study addressing broiler chickens. Hence, the aims of this work were to evaluate the beneficial effects of oridonin supplementation on the growth performance, intestinal microbiota, and gut morphology of broiler chickens and to validate whether dietary oridonin supplementation could attenuate damage to the intestinal lining and protect broilers from negative effects of *S. pullorum*.

## Methods

### Bird husbandry and experimental diets

Three hundred 1-d-old male Arbor Acres (AA) broilers were obtained and divided into five dietary treatments, each with six replicates, 10 chickens per replicate, for a 21-d feeding trial. The 5 treatments were as follows: 1) nonchallenge control chicks (**CON**), 2) chicks treated with Salmonella Challenged Control (**SCC**), 3) chicks treated with *S. pullorum* challenge and 50 mg/kg ORI (O**1**), 4) chicks treated with *S. pullorum* challenge and 80 mg/kg ORI (O**2**), and 5) chicks treated with *S. pullorum* challenge and 100 mg/kg ORI (O**3**). All birds were raised in multi-tiered brooder cages and kept in an environmentally controlled room. During the study period, birds had ad libitum access to water and a balanced unmedicated diet meeting or exceeding the recommendations of the National Research Council (NRC, 1994). The basal (starter) diets were based on corn and soybean meal, as shown in Table 1, and provided as a mash. The experimental design and procedures were approved by the Institutional Animal Care and Use Committee of Nanjing Agricultural University.

**Table 1:** Ingredients and nutrient composition of the basal diet (g/kg diet as-fed basis)

Ingredients (g/kg)	1-21d
Corn	578
Soybean meal (43%, crude protein)	325
Corn gluten meal	30
Soybean oil	27
Limestone	9.5
Dicalcium phosphate	17.5
Salt	3
Choline chloride	3.0
Minerals premix^a^	2.5
Vitamin premix^2^	0.5
L-Lysine HCl	2.5
Methionine	1.5
Total	1000
Calculation of nutrients (g/kg)^3^	
Apparent metabolizable energy (MJ/kg)	12.5
Crude protein	212
Calcium	9.7
Available Phosphorus	4.2
Lysine	10.8
Methionine	4.8
Methionine+cysteine	8.1

Note:^1^Minerals premix provided the following per kg of diet: Fe (ferrous sulfate), 80 mg; Cu (copper sulfate), 8 mg; Mn (manganese sulfate), 110 mg; Zn (Bacitracin Zn), 65 mg; iodine (calcium iodate), 1.1 mg; Se (sodium selenite), 0.3 mg

^2^Vitamin premix provided the following per kg of diet: vitamin A (transretinyl acetate), 10,000 IU; vitamin D_3_ (cholecalciferol), 3000 IU; vitamin E (all-*rac*-*α*-tocopherolacetate), 30 IU; menadione, 1.3 mg; thiamine 2.2 mg; riboflavin, 8 mg; nicotinamide, 40 mg. pyridoxine·HCl, 4 mg; biotin, 0.04 mg; folic acid, 1 mg; vitamin B_12_ (cobalamine), 0.013 mg

^3^The nutrient levels were on an as-feed basis

ORI used in the experiment was purchased from the Laieryin Biological Technology Company Limited (Luoyang, Henan province, P. R. China) with a purity of 98%.

### *Salmonella* infection model

The strain of *S. pullorum* (CVCC 533) was obtained from the China Veterinary Culture Collection Center (Beijing, P. R. China). Broilers in the SCC, O1, O2, and O3 experimental groups were orally treated with 4 × 10^4^ CFU *S. pullorum* bacteria per bird on d 3 posthatch, and the chicks of the CON group were treated with an equal volume of physiological saline.

### Sample collection and procedures

Four, 11, and 18 days after *S. pullorum* infection, one chick per replicate was selected at random, weighed after a 12-h feed restriction, and sent to the Veterinary Laboratory for a bacterial culture of the cecal contents. Subsequently, the broilers were euthanized by cervical dislocation, and an approximately 2-cm-long section of the proximal jejunum was extracted, washed, and fixed for histological examination. The proximal jejunal mucosa was excised carefully, frozen, and kept for further analysis.

### Growth performance

Body weight was measured at 1, 7, 14, and 21 d of age. The amounts of feed supplied and feed waste were also weighed to calculate the feed intake (FI) and feed conversion ratio (FCR). Mortalities were recorded daily to calibrate growth performance parameters.

### Intestinal microbial populations

Approximately 1 g of mixed cecal content was diluted and homogenized. The homogenized suspension was serially diluted in PBS. The samples from the caeca were diluted to10^− 1^, 10^− 2^, 10^− 3^, 10^− 4^, and10^− 5^. From each dilution, 0.1 ml was inoculated on agar plates for aerobics. The dilutions were plated on culture medium. The population of *Lactobacilli* was counted on MRS agar (pH 5.4, Huankai Microbial SCI. and Tech, Co., Ltd. Guangdong, China) after 48 h at 37 °C. The population of *Salmonella* was incubated and counted on bismuth sulfite agar (Qingdao Hope Bio-Technology Co. Ltd., Qingdao, P. R. China) incubated at 37 °C for 24 h. The number of colony forming units (CFU) was expressed as a logarithmic (log_10_) values per gram of intestinal digesta.

### Intestinal histomorphology

Three jejunum cross-sections were prepared using standard paraffin embedding procedures by sectioning at a thickness of 5 μm and staining with hematoxylin and eosin. The jejunum villus height and crypt depth of the stained sections were examined with an Axioplan-2 optical microscope (Carl Zeiss Jena GmbH, Jena, Germany) coupled with a refrigerated QImaging Retiga 4000R digital camera (QImaging, Surrey, British Columbia, Canada) with a charge-coupled device detector, and were expressed in micrometers (μm). A total of 15 complete, well-oriented crypt-villus units were measured with an image processing and analysis system (Version 1, Leica Imaging Systems Co., Ltd., Cambridge, UK) for each type of tissue from each broiler.

### Mucosal antioxidant and immunity index

Approximately 0.3 g of intestinal mucosa samples was homogenized and centrifuged. The supernatant was used for examining mucosal antioxidant and immunity indices. Total superoxide dismutase (T-SOD) activity and malonaldehyde (MDA) content were measured using diagnostic kits (Nanjing Jiancheng Bioengineering Institute, Nanjing, Jiangsu, P.R. China). The levels of immunoglobulin G (IgG) were examined using a commercially available 125 I radioimmunoassay kit with goat anti-chicken IgG (BlueGene, Shanghai, People’s Republic of China). The assay was responsive to a test limit of 0.1 g/mL. The concentration of intestinal immunoglobulin A (IgA, BlueGene, Shanghai, People’s Republic of China) was determined based on the method described by a chicken IgA enzyme-linked immunosorbent assay.

### Statistical analysis

An analysis of variance was performed using the General Linear Model procedure of the Statistical Package for Social Sciences 20.0 (SPSS Inc., Chicago, IL, USA) in a completely randomized design. The differences among all the treatment means were identified using the Tukey’s range test at levels of significance *P* ≤ 0.05.

## Results

### Growth performance and microbial population

Neither *S. pullorum* infection nor ORI supplementation had an effect on FI, BWG, FCR or mortality (Table 2).

**Table 2:** Effect of oridonin on growth performance of broilers challenged with *S. pullorum*

Items	Diet^b^					SEM^c^	*P*-value
	CON	SCC	O1	O2	O3		
BWG^a^, g/bird							
d 1 to 7	95.5	94.8	96.7	104.1	110.6	2.02	0.578
d 8 to 14	189.4	178.8	192.1	195.4	196.4	2.48	0.297
d 15 to 21	336.2	334.8	338.7	342.7	342.9	2.20	0.948
FI^a^, g/bird							
d 1 to 7	113.2	114.7	108.3	114.5	119.5	2.75	0.767
d 8 to 14	276.5	273.5	278.6	277.4	276.9	7.23	0.998
d 15 to 21	556.5	572.6	548.6	552.3	552.2	7.23	0.768
FCR^a^, g feed intake/g weight gain							
d 1 to 7	1.19	1.21	1.12	1.10	1.08	0.03	0.878
d 8 to 14	1.46	1.55	1.45	1.42	1.41	0.05	0.908
d 15 to 21	1.65	1.71	1.62	1.61	1.61	0.03	0.815
Mortality, %							
d 1 to 21	1.14	4.57	2.03	1.49	1.28	1.05	0.583

^a^BWG = Body weight gain; FI = feed intake; FCR = feed conversion ratio

^b^CON = nonchallenge control, SCC = *Salmonella*-challenged control, O**1 =** *S. pullorum* challenge fed the basal diet plus 50 mg/kg ORI; O**2 =** *S. pullorum* challenge fed the basal diet plus 80 mg/kg ORI, O**3 =** *S. pullorum* challenge fed the basal diet plus 100 mg/kg ORI

^c^SEM = standard error of mean

Throughout the evolution of the infection, *Salmonella* reduced the population of *Lactobacillus* at 7 and 14 d and promoted the growth of *Salmonella* colonies in challenged chickens compared with that of the control group (*P* < 0.05) (Table 3). However, ORI supplementation increased the counts of *Lactobacillus* (with the exception of O1 and O2, at 21 d) and reduced the *Salmonella* population (excluding the O1 group, at 21 d) (*P* < 0.05) compared with that of the non-supplemented SCC group.

**Table 3:** Effect of oridonin on cecal microbiota of broilers challenged with *S. pullorum* (log CFU)

Items	Diet					SEM^2^	*P*-value
	CON	SCC	O1	O2	O3		
Day 7							
*L*actobacillus	7.34^a^	5.94^c^	6.59^b^	6.97^ab^	7.40^a^	0.13	0.001
*Salmonella*	7.13^b^	8.24^a^	7.16^b^	7.13^b^	6.94^b^	0.11	0.003
Day 14							
*L*actobacillus	7.41^a^	6.76^b^	7.59^a^	7.73^a^	7.82^a^	0.11	0.002
*Salmonella*	5.64^c^	7.57^a^	6.37^b^	5.72^c^	5.27^c^	0.17	0.002
Day 21							
*L*actobacillus	6.91^b^	6.76^b^	6.83^b^	7.12^ab^	7.39^a^	0.06	0.044
*Salmonella*	6.76^b^	7.67^a^	7.13^ab^	6.69^b^	6.32^b^	0.12	0.005

^1^CON = nonchallenge control, SCC = *Salmonella*-challenged control, O**1 =** *S. pullorum* challenge fed the basal diet plus 50 mg/kg ORI; O**2 =** *S. pullorum* challenge fed the basal diet plus 80 mg/kg ORI, O**3 =** *S. pullorum* challenge fed the basal diet plus 100 mg/kg ORI

^2^SEM = standard error of mean

^3,a,b,c^Values within a row not sharing the same superscript are different at *P* < 0.05; *n* = 6

### Intestinal histomorphology

The SCC group challenged by *S. pullorum* showed a decreased jejunal villus height and V/C value (*P* < 0.05) and an increased jejunal crypt depth at 7 d (*P* < 0.05) compared with those of the CON group (Table 4). ORI supplementation increased the villus height of chickens at all inclusion levels, increased V/C, and decreased crypt depth at 100 mg/kg compared with the values measured for the SCC group (*P* < 0.05). However, the villus height, crypt depth, and V/C value in the intestinal mucosa of the broilers at 14 d or 21 d did not differ among groups.

**Table 4:** Effect of oridonin on the height of villi and crypt depth (μm) in the jejunum of broilers challenged with *S. pullorum*

Items	Diet					SEM^3^	*P*-value
	CON	SCC	O1	O2	O3		
Day 7							
Villus height	1312^a^	1173^b^	1326^a^	1329^a^	1333^a^	17.56	0.007
Crypt depth	245^b^	294^a^	285^ab^	272^ab^	248^b^	5.78	0.004
V/C^1^	5.36^a^	4.00^b^	4.65^ab^	4.89^ab^	5.38^a^	0.74	0.026
Day 14							
Villus height	1228	1185	1314	1357	1362	23.55	0.449
Crypt depth	241	245	242	232	229	8.67	0.984
V/C^1^	5.10	4.84	5.43	5.85	5.95	0.24	0.619
Day 21							
Villus height	1464	1314	1396	1439	1461	45.59	0.744
Crypt depth	191	194	193	190	192	5.16	0.984
V/C^1^	7.66	6.78	7.22	7.57	7.63	0.27	0.185

^1^V/C = Villus height / Crypt depth

^2^CON = nonchallenge control, SCC = *Salmonella*-challenged control, O**1 =** *S. pullorum* challenge fed the basal diet plus 50 mg/kg ORI; O**2 =** *S. pullorum* challenge fed the basal diet plus 80 mg/kg ORI, O**3 =** *S. pullorum* challenge fed the basal diet plus 100 mg/kg ORI

^3^SEM = standard error of mean

^4,a,b^Values within a row not sharing the same superscript are different at *P* < 0.05; *n* = 6

### Antioxidant index

The MDA content in SCC was increased at 21 d (*P* < 0.05) compared with that of CON (Table 5). Moreover, the addition of ORI reduced the MDA content in the jejunal mucosa at 21 d (*P* < 0.05) compared with that of the SCC group. Compared with that of the SCC group, the MDA content in the jejunal mucosa of 7-d-old chicks did not decrease in the O1 or O2 group (*P* > 0.05), but it did decrease in the O3 group (*P* < 0.05). However, there were no differences in MDA content in the jejuna mucosa at 7 or 14 d. The T-SOD activity in jejunal mucosa at 7 d or 21 d did not differ among groups (*P* > 0.05). The T-SOD activity in the SCC group decreased at 14 d (*P* < 0.05) compared with that of CON (*P* < 0.05). The incorporation of 100 mg/kg ORI increased T-SOD activity at 14 d.

**Table 5:** Effect of oridonin on the jejunal mucosa antioxidation function of broilers challenged with *S. pullorum*

Items	Diet					SEM^2^	*P*-value
	CON	SCC	O1	O2	O3		
Day 7							
SOD (unit/mg prot)	230	200	218	230	241	7.49	0.508
MDA (nmol/mg prot)	0.23^ab^	0.29^a^	0.22^ab^	0.21^ab^	0.19^b^	0.01	0.019
Day 14							
SOD (unit/mg prot)	70^a^	63^b^	64^b^	67^ab^	78^a^	1.67	0.021
MDA (nmol/mg prot)	0.24	0.29	0.30	0.24	0.21	0.02	0.052
Day 21							
SOD (unit/mg prot)	184	144	147	164	171	6.16	0.125
MDA (nmol/mg prot)	0.42^b^	0.94^a^	0.53^b^	0.48^b^	0.43^b^	0.04	0.007

^1^CON = nonchallenge control, SCC = *Salmonella*-challenged control, O**1 =** *S. pullorum* challenge fed the basal diet plus 50 mg/kg ORI; O**2 =** *S. pullorum* challenge fed the basal diet plus 80 mg/kg ORI, O**3 =** *S. pullorum* challenge fed the basal diet plus 100 mg/kg ORI

^2^SEM = standard error of mean

^3,a,b,c^Values within a row not sharing the same superscript are different at *P* < 0.05; n = 6

**Table 6:** Effect of oridonin on IgG and IgA contents in jejunal mucosa of broilers challenged with *S. pullorum*

Items	Diet					SEM^2^	P-value
	CON	SCC	O1	O2	O3		
Day 7							
IgG (μg/mgprot)	1.64^b^	2.62^a^	2.07^b^	1.97^b^	1.85^b^	0.08	0.013
IgA (μg/mgprot)	2.43^b^	4.17^a^	3.87^b^	2.77^b^	2.48^b^	0.14	0.021
Day 14							
IgG (μg/mgprot)	2.17^b^	4.28^a^	2.67^b^	2.41^b^	2.19^b^	0.18	0.014
IgA (μg/mgprot)	2.24^b^	4.05^a^	3.76^b^	2.42^b^	2.21^b^	0.22	0.011
Day 21							
IgG (μg/mgprot)	2.42^b^	5.07^a^	3.45^b^	2.87^b^	2.68^b^	0.23	0.003
IgA (μg/mgprot)	2.05^b^	2.41^a^	1.92^b^	1.74^b^	1.54^b^	0.21	0.011

^1^CON = nonchallenge control, SCC = *Salmonella*-challenged control, O**1 =** *S. pullorum* challenge fed the basal diet plus 50 mg/kg ORI; O**2 =** *S. pullorum* challenge fed the basal diet plus 80 mg/kg ORI, O**3 =** *S. pullorum* challenge fed the basal diet plus 100 mg/kg ORI

^2^SEM = standard error of mean

^3,a,b,c^Values within a row not sharing the same superscript are different at *P* < 0.05; n = 6

### IgA and IgG concentration

*Salmonella* infection increased the concentrations of jejunal IgA and IgG at 7, 14, and 21 d (*P* < 0.05) (Table 6). Dietary ORI decreased jejunum IgA and IgG contents at 7, 14, and 21 d (*P* < 0.05) compared with those of the SCC (*P* < 0.05). However, there were no differences in the concentration of jejunal IgA or IgG between the CON and ORI group at 7, 14 or 21 d (*P* > 0.05).

## Discussion

*Salmonella* infections resulting in slowed production performance, intestinal colonization, inflammation responses, and invasion of internal organs in broiler chickens have been reported [7]. In the present study, *Salmonella* infection had no effect on the production performance of the chicks, but certain short-term symptoms, such as reluctance to move, emotional panic, lethargy, lack of appetite, and decreased water intake appeared at 6 d of age, after the first inoculation. There were no apparent clinical symptoms at 11 or 18 d. This finding is consistent with that of Chen et al. (2015), who noted that clinical symptoms vanished after operative treatment [8]. However, in some studies, the effects of salmonella were more severe likely due to age, bacterial serotype, dose or environmental conditions [3]. In any case, ORI did not show any effects on productive performance likely because of the lack of an effect of infection on such variables.

In previous studies, *Salmonella* caused a high level of infection in the caecum [9] and decreased the number of *Lactobacillus* colonies [10]. In the present study, the *Salmonella* count in SCC groups was higher than that of uninfected groups, whereas *Salmonella* inhibited the growth of *Lactobacillus*. Furthermore, *Lactobacillus* numbers markedly increased and *Salmonella* numbers decreased in the ORI treatment, with values similar to those of the control group. These results indicate that ORI can counteract *Salmonella* infection, promoting the growth of *Lactobacilli* to levels comparable to those in healthy chickens [11].

Villus height and crypt depth are important indices of the functional capacity of the enterocytes of broilers, and the VH:CD ratio has been shown to immediately affect the digestion and absorption of the intestinal mucosa [12]. In the current study, nearly all measured morphometric parameters of the jejunum were affected after 4 d post infection. This finding agrees with the observation that *Salmonella* leads to apoptosis of cells in the jejunum [13]. Such processes tend to be attributed to the impairment of epithelial protein synthesis and function after *Salmonella* infection, which dramatically influence the early intestinal morphological development of chickens. As previously noted, dietary inclusion of ORI resulted in increased villus height and VH:CD ratio and reduced crypt depth in broilers. The development of jejunal villi may be enhanced by the antibacterial and anti-inflammation properties of ORI.

Some studies have shown that intestinal oxidative stress and substantially impaired mucosal barriers occur in *Salmonella* challenged broilers [8]. The results of this study showed that *Salmonella* infection led to an imbalance of cytosolic redox status in favor of prooxidants, causing intestine cells to experience a state of oxidative stress in infected broilers. The results demonstrate that intestinal mucosal barriers were substantially disrupted in *Salmonella* challenged broilers. The molecular mechanisms of this mucosal barrier injury are completely unknown but may be due to the strong oxidative burst against *Salmonella* infection. However, administration of ORI to infected animals has maintained cellular antioxidant defense systems (T-SOD) at their normal levels, which might be due to a reduction in oxidant lesions by regulating the oxidation of T-SOD enzymes in the intestinal immune response.

The small intestine is one of the most important parts of the mucosal immunity system and provides antigen-specific protection by producing antibodies. Certain bacteria and viruses affect immunoglobulin synthesis, and *Salmonella* could induce marked intestinal immune responses characterized by the secretion of a large number of immunoglobulins in animals [14, 15]. Thus, we investigated the effects of *Salmonella* challenge on immunoglobulins in the jejunum of broilers. The results verified that SCC chicks experienced increased concentrations of jejunal IgA and IgG in response to *Salmonella* intestinal infection.

IgA and IgG, the major immunoglobulins produced by lymphocytes of the mucosa, are clearly involved in the development of the intestinal immune response to *Salmonella* and are critical for protecting mucosal surfaces against toxins, viruses, and bacteria by neutralizing them or preventing them from binding to the mucosal surface [16–18]. In the present study, we found that ORI can decrease immunoglobulin concentrations (IgA and IgG) in the jejunum. The mechanisms of ORI’s immune-protecting properties may be partially attributed to T cell depletion in the peripheral immune system [19]. Additional studies will be required before the potential applications of ORI in feeding practices are fully understood.

## Conclusions

In conclusion, our results suggest that *S. pullorum* can reduce the counts of *Lactobacillus* and the villi height of the small intestine and increase the counts of *Salmonella*, IgA concentrations in the jejunum, and the MDA content in the jejunum. ORI could protect the intestinal epithelium and normalize bacterial populations and immune response in *Salmonella* challenged broilers.

## Abbreviations

BWG: Body weight gain
**CON**: Nonchallenge control
FCR: Feed conversion ratio
FI: Feed intake
IgA: Immunoglobulin A
IgG: Immunoglobulin G
MDA: Malonaldehyde
ORI: Oridonin
**SCC**: *Salmonella pullorum* Challenged Control
T-SOD: Total superoxide dismutase
